# Supplementary figures and images for: Sequence variation and immunogenicity of the Mycoplasma genitalium MgpB and MgpC adherence proteins during persistent infection of men with non-gonococcal urethritis
Source: PLoS One. 2020 Oct 12;15(10):e0240626. doi: 10.1371/journal.pone.0240626 (PMC7549776; doi:10.1371/journal.pone.0240626)

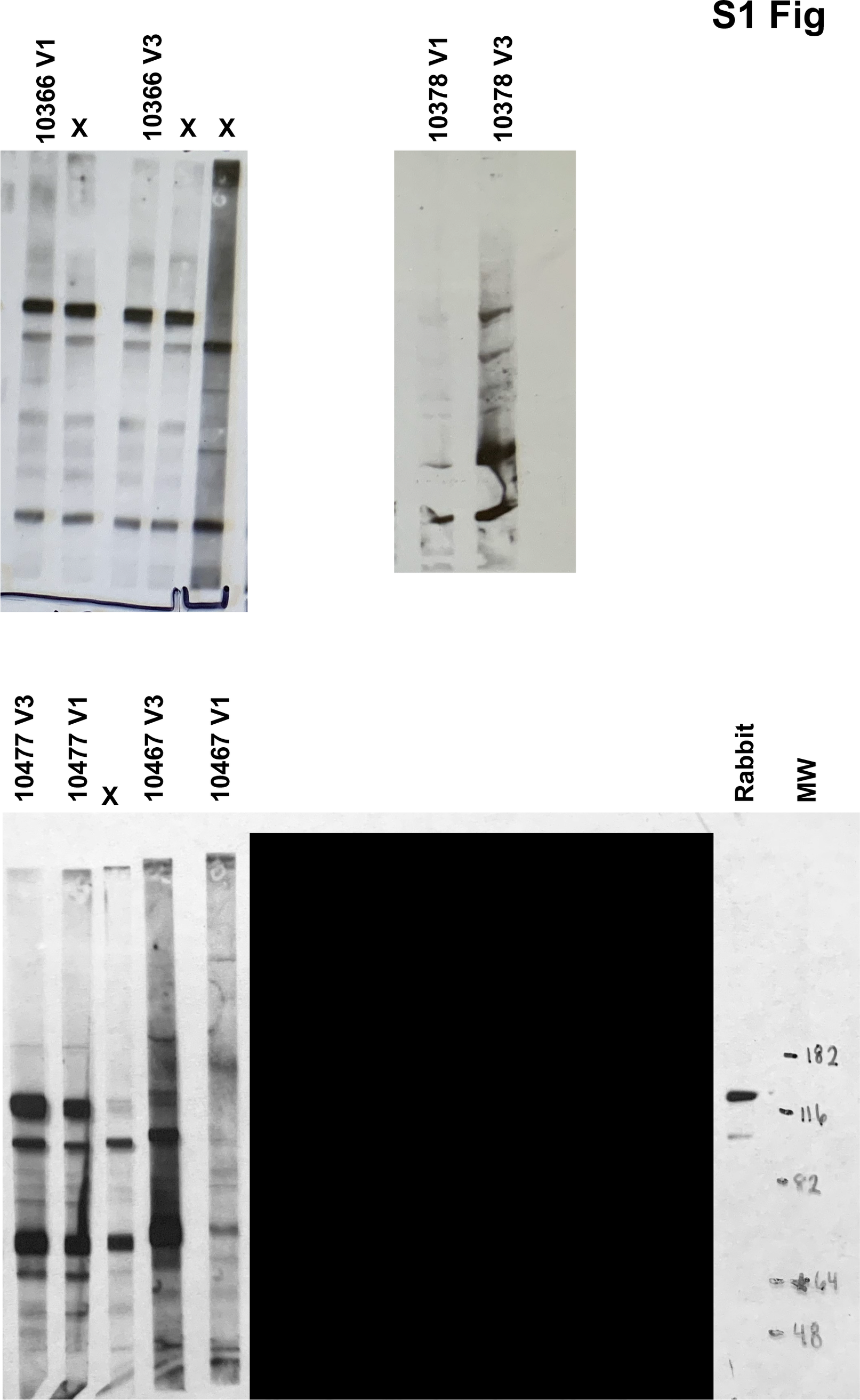

Supplement: S1 Fig — Original blots showing reactivity of patient sera to M. genitalium whole cell lysates. (TIF) [file pone.0240626.s001.tif]
